# Naturally Fermented Acid Slurry of Soy Whey: High-Throughput Sequencing-Based Characterization of Microbial Flora and Mechanism of Tofu Coagulation

**DOI:** 10.3389/fmicb.2019.01088

**Published:** 2019-05-14

**Authors:** Yunhe Xu, Qing Ye, Huajiang Zhang, Yang Yu, Xiaona Li, Zhen Zhang, Lili Zhang

**Affiliations:** ^1^Department of Food Science and Engineering, Jinzhou Medical University, Jinzhou, China; ^2^Department of Food Science, Northeast Agricultural University, Harbin, China; ^3^Department of Food Science, Shenyang Agricultural University, Shenyang, China

**Keywords:** coagulation, *Lactobacillus casei*, tofu processing, soy protein whey, naturally fermented acid slurry

## Abstract

Tofu processing generates large quantities of whey as waste water. Although naturally fermented whey serves as a coagulant, the critical constituents remain unknown. High-throughput sequencing identified predominant *Lactobacillus* in the naturally fermented acid slurry. *Lactobacillus casei* YQ336 with high coagulating ability and lactic acid production was isolated and its soy protein coagulating mechanism was determined. The acid in YQ336 fermented acid slurry lowered soy milk pH and reduced negatively charged groups of denatured soy protein, leading to coagulation. Acid slurry metal ions also promoted pH decline; moreover, YQ336-produced protease might partially hydrolyse soy protein, further promoting coagulation. Thus, organic acids, metal ions, and enzymes together promote coagulation, with the former acting as the main contributing factor. This study will pave the way for future industrial application of *L. casei* YQ336 in acid slurry tofu processing and food manufacturing, thereby potentially reducing resource waste and environmental pollution.

## Introduction

Tofu, a traditional Chinese food also known as soy curd, constitutes an excellent source of plant protein that is rich in beneficial lipids, vitamins, minerals, and other bioactive compounds including isoflavones and soyasaponin. Epidemiological studies indicate that tofu consumption reduces the risk of many chronic diseases, such as cancer and cardiovascular diseases (Jacobsen et al., 1998; Ginting et al., 2003; Wang and Mejia, 2005), supporting tofu as the most popular soy food in Eastern diets (Qiao et al., 2010).

However, large quantities of tofu whey are produced during tofu processing that are often discharged into the environment as waste water (Yu et al., 2015). Moreover, the abundant nutritious and functional factors in tofu whey, such as soluble proteins, lipids, soluble sugar, vitamins, minerals, oligosaccharides, isoflavone, and saponin, support the growth of numerous microorganisms, causing serious environmental pollution (Masangkay, 2012; Corzo-martínez et al., 2016; Li et al., 2017). Alternatively, the acid slurry produced by tofu whey naturally fermented by its normal micro biota has been used as a traditional tofu coagulant for 400 years in China (Qiao et al., 2010; Fei et al., 2018; Zhang et al., 2018). Tofu produced with natural acid slurry is more acceptable and enjoyed as a staple food owing to its nice flavor, light sweet taste, and good texture (Fei et al., 2018). Furthermore, the use of fermented tofu whey in tofu processing avoids the environmental pollution caused by tofu whey emissions and serves as a tofu coagulant to avoid the addition of chemical coagulants, such as calcium sulfate or chloride, thus being conducive to the production of greener and safer tofu products.

Because the acid slurry is a product of natural fermentation, its quality is greatly affected by environmental factors such as region, season, temperature, and human factors, resulting in an instability of tofu quality and flavor (Qiao et al., 2010; Fei et al., 2018; Zhang et al., 2018). In turn, acid slurry quality, which depends on the fermented microorganisms, is directly related to tofu quality from the perspective of intact food processing (Rekha and Vijayalakshmi, 2013; Nguyen, 2014; Fei et al., 2018). Microorganisms in naturally fermented acid slurry include both beneficial bacteria such as lactic acid bacteria and harmful spoilage bacteria such as *Escherichia coli*. To promote the rapid predominance of tofu-coagulation microorganisms, acid slurry is traditionally maintained by inoculating fresh tofu whey with a small volume of previously fermented acid whey (Qiao et al., 2010).

The microorganisms from acid slurry in different regions have been studied by conventional plate culture methods. In an acid slurry sample naturally fermented in the laboratory, Qiao et al. (2010) showed that lactic acid bacteria predominated during acid slurry fermentation. In comparison, Enterobacteriaceae, yeast, and mold remained below the detectable threshold, whereas spore-forming bacteria and acetic acid bacteria slowly increased over fermentation time. Alternatively, Fei et al. (2018) studied acid slurry samples from Jiangmen City, Guangdong Province by high-throughput sequencing. They found that *Lactobacillus* was the predominant genus (95.31%) in an acid slurry, whereas *Picha*, *Enterococcus*, *Bacillus*, and *Acetobacter* were present at only trace levels (0.90, 0.04, 0.02, and 0.09%, respectively). Therefore, although the differences in manufacturing technology and geographical location may affect microflora diversity in the acid slurry, lactic acid bacteria comprise the predominant bacteria and mainly determined acid slurry chemical changes. To date, *Lactobacillus acidophilus*, *Lactobacillus plantarum*, *Lactobacillus amylolyticus*, and *Leuconostoc mesenteroides* have been isolated from naturally fermented acid slurry in different regions (Qiao et al., 2010). However, no industrial strains are currently suitable for acid slurry fermentation.

Three main mechanisms have been proposed for explaining the coagulation of tofu: salt, acid, and enzyme coagulation (Kohyama et al., 1995; Obatolu, 2008; Ono, 2008; Murekatete et al., 2014a; Zhu et al., 2016; Zheng et al., 2018). Organic acids produced by lactic acid bacteria fermentation induce tofu coagulation and are considered the primary factor underlying this phenomenon (Qiao et al., 2010; Wang et al., 2013). However, naturally fermented acid slurry also contains inorganic salts derived from soy and processing water, along with enzymes produced by microbial metabolism. Although salts and enzymes can also coagulate tofu, their role in acid slurry-mediated tofu coagulation remains unknown.

To determine advantageous microbes for promoting soy protein coagulation and the underlying mechanisms in acid slurry, in this study acid slurry naturally fermented from Shandong, Jiangsu and in the laboratory was first subjected to high-throughput sequencing and oligotyping technology combined with traditional culture methods to analyze the microbial flora. To screen the strains suitable for industrial fermentation of acid slurry, the tofu coagulation ability of acid slurry fermented by the identified strains was determined along with the underlying. This study will lay a foundation for standardized acid slurry tofu production and improving soybean resource utilization efficiency.

## Materials and Methods

### Samples and Medium

The acid slurry samples were obtained from Jianiang Tofu Square, Linyi City, Shandong Province, China (LY), Huanglaoda Acid Slurry Tofu Square in Jiangsu Province, China (RGBJ) (both samples transferred to the laboratory with ice), and by natural fermentation in the laboratory (NFAS). Microorganisms were plated for enumeration and isolation on the same day. Phosphate-buffered saline (PBS, pH 7.2) was purchased from Sigma Chemical, Co. (St. Louis, MO, United States). All other chemical reagents were of analytical grade. Soy whey medium comprised 2% glucose added to fresh tofu whey, sterilized by autoclaving at 115°C for 15 min.

### Tofu Preparation

The tofu was prepared previously described with some modifications (Rekha and Vijayalakshmi, 2013; Zhu et al., 2016). The soy beans were soaked in distilled water at 25°C for 10–12 h, washed, then beaten at a ratio of 1:10 to water. The slag was filtered through a 100 mesh nylon sieve to obtain soy milk, which was boiled for 5 min using an electromagnetic oven (Media Co. Ltd., Foshan, China). When the soy milk temperature dropped to 80–90°C, the coagulant was slowly poured into a steel container while stirring (for around 5 min), with the acid slurry addition stopped upon soy milk coagulation. The coagulated soy milk was allowed to stand undisturbed for 15 min to ensure complete coagulation. The curd was pressed into a tofu mold (12 cm × 12 cm × 8 cm depth) for 30 min using special 4 kb barrel to obtain the final product. Tofu whey was used as a raw material for subsequent naturally fermented acid slurry and lactic acid bacteria culture medium. The resulting tofu was stored at 4°C until analysis.

### Preparation of Acid Slurry by Natural Fermentation in the Laboratory

Initially, tofu was made without acid slurry. White rice vinegar (pH 3.5, Foshan Haitian Flavouring & Food Co., Ltd., China) diluted with distilled water to pH 4.0 was used instead of the acid slurry as a tofu coagulant. In this process, a large amount of tofu whey was generated and mixed with the remaining coagulant at 3:1 v/v. Natural fermented acid slurry was used as coagulant when its pH had been lowered to 4.0 after fermentation at 30°C. During subsequent fermentation, the large amount of tofu whey generated was mixed with the remaining coagulant at 3:1 v/v. The naturally fermented soy whey could be used as coagulant when its pH lowed to 4 at 30°C. It was usually activated once every 3 days; after a total of six fermentations, it was used as the naturally fermented acid slurry sample for later experiments (Qiao et al., 2010; Fei et al., 2018).

### Microorganism Counts

Microorganisms were enumerated by serial dilution and plating. Bacterial counts and isolation were conducted on nutrient agar medium supplemented with cycloheximide (50 μg/mL) to inhibit fungal growth (Laureys and De Vuyst, 2014; Zhang et al., 2017); plates were incubated at 37°C for 24 h. Yeasts and molds were inoculated on Rose Bengal agar plates, incubated at 25°C for 3–5 days (Zhang et al., 2017), and distinguished by morphology: smooth and wet colonies were considered as yeasts; downy or furry colonies were considered to be molds. Bacterial colonies were counted using automatic colony counters (Papalexandratou et al., 2011; Zhang et al., 2017).

### Microbial Flora Analysis by High-Throughput Sequencing Technology

Microbial genomic DNA was extracted from 1 mL acid slurry using the Tiangen DNA stool mini kit (cat#DP328, Beijing, China) according to manufacturer instruction. The 16S rDNA V4 variable region was amplified using the universal primers 520F (5′-AYTGGGYDTAAAGNG) and 802R (5′-TACNVGGGTATCTAATCC) (Zhang et al., 2017). PCR amplification and sequencing library construction were performed as described by Xu et al. (2016). For each sample, barcoded V4 PCR amplicons were sequenced using the Illumina MiSeq platform (Ma et al., 2015). 16S rDNA V4 variable region amplification and sequencing was completed by Personal Biotechnology, Co., Ltd. (Shanghai, China). Sequence reads excluded from analysis included those <150 bp in length, average Phred score < 20, containing ambiguous bases, homopolymer run > six bases, or mismatched to primers. Sequences that passed quality filtering were assembled using Flash^1^, which required ≥10 bp read overlap, without any mismatches. The reads that could not be assembled were discarded. Chimera sequences were removed using UCHIME in mothur (version 1.31.2 2^2^).

Sequence clustering was performed using the UCLUST algorithm in QIIME^3^; the sequences were clustered into operational taxonomic units (OTUs). The longest sequence in each cluster was selected as the representative. The taxonomy of each OTU was assigned by BLAST-searching the representative sequence against the Greengenes reference database (Release 13.8 4^4^) (Shu et al., 2015; Xu et al., 2016).

Reads assigned to the *Lactobacillus* genus were extracted and an entropy analysis and oligotyping were carried out as described by the developers (Eren et al., 2013). After the initial round of oligotyping, high entropy positions were chosen (−C option): 11, 12, 13, 14, 15, 35, 36, 42, 48, 49, 53, 68, 69, 70, 71, 72, 91, 135, 159, 169, 220, and 221. To minimize the impact of sequencing errors, we required an oligotype to be represented by at least 10 reads (−M option). Moreover, rare oligotypes present in less than three samples were discarded (−s option). These parameters led to 121,222 (93.81%) sequences remaining in the dataset. BLASTn was used to query the representative sequences against the NCBI nr database, and the top hit was used for taxonomic assignment (Giello et al., 2018).

### Isolation of Coagulating Strains

The bacteria and yeasts in the acid slurry sample were separated and purified on MRS and Rose-Bengal media, respectively, using the plate coating method. The acid slurry samples were diluted to 10^−7^ by a 10-fold gradient dilution method, and 0.1 mL of 10^−5^, 10^−6^, and 10^−7^ sample dilutions were applied to MRS medium containing 2% CaCO_3_, and 0.1 mL of 10^−3^, 10^−4^, and 10^−5^ dilutions applied to the Rose-Bengal medium, then incubated for 48 h at 37°C (Manini et al., 2015; Kumari et al., 2016). The bacterial colonies with larger karst caves and different forms of yeast colonies were selected, then separated and purified via 2–3 slab scribing procedures. The purified strains were inoculated in soy whey medium and cultured at 37°C for 24 h; strains with strong coagulating ability were selected (Mahmoudi et al., 2016). The screened strains were inoculated in soy whey medium for 24 h, then the lactic acid content of the fermentation liquid was determined by high performance liquid chromatography (HPLC). The strains with more lactic acid production were selected for strain identification.

### Identification by 16S rDNA Sequencing

Pure isolates were grown to a late stationary phase in 5 mL of medium, then centrifuged (10 min, 4,000 × *g*). Each cell pellet was resuspended in 0.5 mL dH_2_O, then DNA was extracted using the Tiangen DNA stool mini kit. Full-length 16S rDNA amplicons were generated using bacterial primers 27F (5′-AGA GTT TGA TCC TGG CTC AG) and 1492R (5′-CTA CGG CTA CCT TGT TAC GA). The PCR thermocycling conditions were as follows: an initial denaturation at 95°C for 5 min, 35 cycles of 95°C for 30 s, 58°C for 30 s, and 72°C for 90 s, and a final extension step of 72°C for 7 min. PCR products were purified and sequenced using an ABI 3730 automated sequencer at Personal Biotechnology Co., Ltd. (Shanghai, China).

All sequences were matched against similar 16S rDNA nucleotide sequences in GenBank using the BLASTN program ^5^ (Altschul et al., 1997). Bacterial identification was assumed when the query sequence showed >97% similarity with the target 16S rRNA gene sequence (Gevers et al., 2005; Guo et al., 2010).

The 16S rDNA sequences were aligned using CLUSTAL W and a phylogenetic tree was constructed using the neighbor-joining method in PHYLIP. Bootstrap resampling was carried out with 1,000 replications to estimate the confidence of the tree topologies.

### Determination of the Growth and Acid Production Curve of *L. casei* YQ336

One loop of *L. casei* YQ336 cells was picked from soy whey slant medium, inoculated into 5 mL soy whey medium and cultured at 37°C for 24 h, then inoculated into soy whey medium at 3% inoculation and cultured at 37°C, sampling at intervals of 4 h. The viable cell count and the total acid yield were measured, and a curve was drawn.

### Determining the Effect of Physical and Chemical Treatments on the Coagulating Ability of Acid Slurry Fermented by *L. casei* YQ336

To prepare acid slurry fermented by *L. casei* YQ336, *L. casei* YQ336 culture was grown as described in the previous section and cultured at 37°C for 48 h in soy whey medium. Then, 100 mL of *L. casei* YQ336 fermentation broth was centrifuged at 10,000 × *g* for 20 min to obtain a supernatant and a bacterial cell precipitate. The supernatant was used as a coagulant to measure its coagulation activity on the soy protein. The precipitated cells were dissolved in 100 mL deionised water to measure the coagulation activity of the mixed solution on the soy protein. *L. casei* YQ336 fermentation broth (100 mL), boiled for 20 min to destroy the enzymes in the metabolite, was used to examine the effect of the enzyme on the coagulation activity (Zhang et al., 2017). The pH of the *L. casei* YQ336 fermentation broth was adjusted to 7.0 with 1 mol L^−1^ NaOH to determine the effect of pH on its coagulation activity.

### Composition Analysis of Acid Slurry Fermented by *L. casei* YQ336

*Lactobacillus casei* YQ336 was inoculated to the soy whey medium at an inoculation of 3% and cultured at 37°C for 48 h, then the following indicators of the fermentation broth were determined. The ash content in the acid slurry was determined using the burning method. The direct drying method was used to determine the moisture content in the acid slurry, whereas the Soxhlet extraction method was used to determine the fat content. The protein content and soluble solids in the acid slurry were determined using the Kjeldahl method (Zhao and Huang, 2016) and the refractive method (Liu and Lei, 2016), respectively.

### Determination of Metal Ions in Acid Slurry Fermented by *L. casei* YQ336

Determination of the main metal ions Ca^2+^, Mg^2+^, Na^+^, and K^+^ in the acid slurry fermented by *L. casei* YQ336 was performed using an inductively coupled plasma optical emission spectrometer (ICP-OES) as follows. The standard solution was configured, with each element diluted to 20 mg ⋅ L^−1^ stock solution with 5% HNO_3_, then this was further diluted to 200 and 400 μg ⋅ L^−1^ stock solutions, with these subsequently prepared into a series of standard solutions of 0, 8, 16, 24, 32, 48, and 64 μg ⋅ L^−1^. A standard curve was drawn according to the PerkinElmer Win Lab 32 for ICP software method. The optimum wavelength of each element was selected and the standard solutions were sequentially detected on the machine. The detection limit was determined from the resulting standard curve. The linear correlation coefficient was greater than 0.99.

For determination of metal ions, 0.2–1 g sample was weighed into the polytetrafluorethylene digestion tank (accurate to 0.1 mg), 5 mL of nitric acid was added, and the mixture allowed to stand. After completion of the reaction, the lid was sealed and placed in a microwave digestion apparatus for digestion. The digestion procedure was held at 100, 140, 160, and 180°C for 3 min each, and finally at 190°C for 15 min. After the temperature had cooled to below 50°C, the digestion tank was moved into a fume hood, opened, and the sample was rinsed with ultrapure water, transferred to a 50 mL volumetric flask, rinsed at least 3–4 times, and diluted with ultrapure water. The volume was fixed to the scale, recorded as *C*_1_ to be tested, with the blank control recorded as *C*_0_. If the sample was not completely dissolved and had impurities, it was passed through a 0.45 μm aqueous filter prior to use (Khan et al., 2014).

ICP-OES calculation formula:

(1) $$X=\frac{(c_{1}-c_{0})\times \text{V}\times \text{f}\times 100}{\text{m}\times 1000}$$

where *X* is the content of the element in the sample (mg⋅100g^−1^), *C*_1_ is the concentration of the elements in the sample solution (g⋅mL^−1^), *C*_0_ is the concentration of the elements in the blank (μg mL^−1^), *V* is the sample volumetric volume (mL), f is the dilution factor, and m is the sample quality (g).

### Determination of Organic Acid in Acid Slurry Fermented by *L. casei* YQ336

The organic acids (tartaric, lactic, acetic, fumaric, citric, succinic, and oxalic acids) were measured using HPLC (Agilent, Santa Clara, CA, United States) equipped with an XSelect HSS T3 analysis column (250 mm × 4.6 mm, 5 mm particle size, Agilent). A 10 mL aliquot of fermented tofu whey was centrifuged (14,000 × *g*, 10 min), then filtered using 0.22 μm pore size membrane into an HPLC vial. Separated conditions used an isocratic flow rate of 0.8 mL min^−1^ of 2% acetonitrile-98%H_3_PO_4_ (pH 2.0), which had been filtered through a 0.45 μm membrane and degassed via ultrasonication (50°C, 20 min). Detection was monitored at 210 nm. Column oven temperature was maintained at 35°C. The concentration of each organic acid standard sample was diluted to 0.5, 1, 2, 3, and 4 mg ⋅ mL^−1^ and stored at 4°C. Three measurements were made for each sample and averaged (Kelebek et al., 2009; Morales-de la Peña et al., 2011; Ec et al., 2016).

### Effect of Simulated Acid Slurry on the Coagulation of Soy Protein

According to the main components of the acid slurry fermented by *L. casei* YQ336, the main organic acid and metal ion configuration was added in proportion to deionised water to obtain simulation solutions. The first simulated acid slurry comprised a ratiometrically prepared organic acid and metal ion solution. The pH of the simulated solution was adjusted to 3.6 with sodium hydroxide or potassium hydroxide. The second simulated acid slurry comprised a solution containing only the same proportion of metal ions. The third simulated acid slurry was a solution containing only organic acids.

Using *L. casei* YQ336 fermented acid slurry as a control, the simulated solutions were used to make tofu and the coagulation, pH prior to and following the point of detection of the soy protein, and tofu texture were observed; the tofu was also analyzed by scanning electron microscopy (SEM).

### Texture Properties of Tofu

For tofu sensory evaluation, the color, smell, tissue state, taste, and cross-sectional structure were scored according to the percentage system by 10 teachers and students of food science and engineering as previously described (Kamizake et al., 2018). The scoring criteria are shown in Supplementary Table S1.

### Mechanical Properties and Cookability

Tofu texture was determined using a physical property tester (Model TA.XT2i, Stable Micro Systems, Godalming, United Kingdom). The penetration test was performed using TPA measurement mode, cylindrical probe (25 mm diameter), test speed of 60 mm/min, starting point induction force of 1 N, deformation rate of 40%, compression frequency: 2 times, and interval of 0.5 s (Cao et al., 2017).

To determine cookability, the tofu was cut into 2.0 cm × 3.0 cm × 2.0 cm squares and placed in boiling water for 1.5 h. The integrity and ability to be lifted of the test piece were examined, and the occurrence, time, and condition of breakage were recorded (Shen et al., 2010; Kamizake et al., 2018).

### Microstructure Analysis of Tofu

The tofu was cut into 1.0 mm thick, 3.0 mm long, and 3.0 mm wide pieces, fixed with a glutaraldehyde solution [3% (v/v) in 0.01 M phosphate buffer, pH 7.2] on brass stubs, and chromium-coated using a Xenosput 2000 chromium coater with the deposition parameters of 0.06 sputter Amps for 40 s. Coated preparations were visualized by Hitachi S4800 SEM (Hitachi Ltd., Tokyo, Japan) at an accelerating voltage of 2 kV (Zhu et al., 2016; Zhang et al., 2017).

### Statistical Analysis

Data were obtained in triplicate and are reported as averages; statistical analyses were performed to determine significant differences (*p* < 0.05) among obtained results using ANOVA followed by Duncan’s multiple range test. All data were analyzed using SPSS 16 software (SPSS, Chicago, IL, United States).

## Results and Discussion

### Microbial Composition in Naturally Fermented Acid Slurry

In the manufacture of tofu, soy milk is pressed to produce substantial amounts of soy whey containing soluble carbohydrates, a small amount of protein, and other nutritional factors. Thus, soy whey comprises a good culture medium for growth of various bacteria (Yu et al., 2015), with the microorganisms in the resulting acid slurry directly related to its quality and tofu coagulation ability (Qiao et al., 2010; Fei et al., 2018). In the present study, plate colony counting methods were used to detect the number of bacteria, yeasts, and molds in naturally fermented acid slurry from three different sources. Table 1 shows that high bacteria counts were obtained (≥6.52 ± 0.13 log CFU mL^−1^) from all three acid slurry sources, whereas yeasts were <4.13 ± 0.10 log CFU mL^−1^ with even fewer molds, all being <2 log CFU mL^−1^. Bacteria, therefore, were predominant in naturally fermented acid slurry.

**Table 1:** Microbial counts (log CFU mL^−1^) of the three sources of acid slurry.

Sample of acid slurry	Bacteria	Yeast	Mold
LY	7.35 ± 0.25^b^	4.39 ± 0.05^b^	<2
RGBJ	6.52 ± 0.13^a^	4.13 ± 0.10^a^	<2
NFAS	6.82 ± 0.06^a^	4.19 ± 0.06^a^	<2

*The unit of microbial count for acid slurry is log CFU mL^−1^. Results are given as the means ± SD of triplicate determinations. Values with different small letters within the same column are significantly different (p < 0.05).*

**FIGURE 1:**
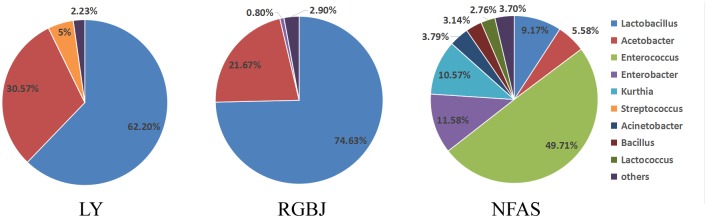
Distribution of bacteria in the acid slurry at the genus level.

To further investigate the predominant bacteria in naturally fermented acid slurry, the bacterial composition was evaluated by high throughput sequencing of the 16S rRNA gene V4 regions. The bacterial community was analyzed at the genus level by comparison with the Greengenes reference database. *Lactobacillus* was the predominant bacteria in the naturally fermented acid slurry, accounting for 49.73–74.63% (Figure 1). This is consistent with previous findings from both high-throughput sequencing (Fei et al., 2018) and plate colony counting (Qiao et al., 2010) analyses of laboratory-made naturally fermented acid slurries. Li et al. (2017) reported that 112 *Lactobacillus* spp., 18 *Streptococcus* spp., and 33 yeasts were isolated and identified from naturally fermented soy whey (NFSW). *Lactobacillus* spp. were found to be the major effective strains, and seven *Lactobacillus* strains produced enough acid to coagulate soy milk. Fei et al. (2018) investigated the microbial diversity of natural fermented tofu whey (NFTW) with high-throughput sequencing. *Lactobacillus* (95.31%) was the predominant genus in the microbial community of NFTW, whereas *Picha, Enterococcus, Bacillus*, and *Acetobacter* represented about only 0.90, 0.04, 0.02, and 0.09% of the genera, respectively. At the species level, nine species of *Lactobacillus* were identified based on assembled 16S rDNA sequences. Compared with the culture method results, only *L. amylolyticus*, *Lactobacillus mucosae*, *Acetobacter pasteurianus*, and *Bacillus cereus* were identical, which shows the limitations of both methods (Fei et al., 2018).

However, the *Lactobacillus* proportion of NFAS was lower than that of LY and RGBJ. Environmental factors may in part account for the difference. In addition, LY and RGBJ were obtained from the tofu factory after several years or even decades of repeated fermentation, whereas NFAS was only obtained after six fermentation rounds for 18 days. Thus, *Lactobacillus* enrichment in NFAS was less than that in LY and RGBJ. Nevertheless, *Lactobacillus* appears to be the predominant genus in naturally fermented acid slurry regardless of origination from different manufacturers or the laboratory.

### Isolation and Identification of Soy Protein Coagulating Strains From Naturally Fermented Acid Slurry

As *Lactobacillus* represented the predominant bacteria in naturally fermented acid slurry, lactic acid bacteria were isolated using MRS medium, with a small number of yeasts and molds also isolated using Rose-Bengal medium. In total, 59 bacteria and 16 yeasts were isolated from the acid slurry; these were then inoculated and cultured in soy whey medium to determine their coagulating abilities. Five isolates, all bacterial strains, exhibited coagulating ability; of these, four strains were identified as *Lactobacillus* (*L. casei* YQ116; *L. pentosus* YQ127; *L. casei* YQ336; *L. pentosus* YQ338) and one as *Enterococcus* (*Enterococcus faecium* YQ337) by 16S rDNA sequence homology comparisons. Conversely, the yeasts did not show any coagulating ability, indicating that *Lactobacillus* constituted the predominant coagulating microorganism in the acid slurry.

*Lactobacillus* identification at the species level was achieved by oligotyping, which decomposes a given taxon into high-resolution units (oligotypes) by considering the nucleotide positions identified to be the most information rich (Eren et al., 2013). This approach enables the detection of ecologically meaningful differences within a single genus in gut and environmental microbiota (Koskey et al., 2014; Eren et al., 2015; De Filippis et al., 2016). We carried out oligotyping of *Lactobacillus* sequences. Among 486 different oligotypes identified, 24 oligotypes showed a relative abundance greater than 2% (at least in one sample). BLASTn identification revealed two species (*Lactobacillus delbrueckii* and *L. amylolyticus*). The results show that the *Lactobacillus* oligotype pattern in the RGBJ sample was clearly different from that of the other two groups, revealing fine-scale biogeographical patterns between closely related *Lactobacillus* strains (Supplementary Figure S1). Through sequence alignment, we found that four strains with coagulating ability belong to the dominant genus of *Lactobacillus* in the acid slurry, but, by oligotyping, we showed that they did not belong to the dominant *Lactobacillus* oligotypes (>2%). Of course, this may be related to selection in the culture medium, limitations of screening conditions, or the plate separation technique itself. Therefore, based on oligotyping, further study of the flora composition and acid-producing lactic acid bacteria distribution in acid slurries is needed.

The five identified strains of lactic acid bacteria with coagulating ability were inoculated into soy whey medium, cultured at 37°C for 24 h, and the lactic acid production in the fermentation liquid was determined by HPLC. Among these, strain YQ336 produced the highest production of lactic acid, reaching 15.69 ± 0.24 g L^−1^. Therefore, YQ336 was selected as the fermentation strain for the subsequent analysis of acid slurry fermentation. Following culture in soy whey agar medium at 37°C for 48 h, YQ336 colonies presented as milky and round with a smooth and opaque surface and diameter of 0.8–1.2 mm. The cells of the strain YQ336 comprised Gram-positive rods arranged in a chain with no spore or flagellum. Physiological and biochemical characteristics of strain YQ336 are shown in Table 2.

**Table 2:** Physiological and biochemical characteristics of strain YQ336.

Experimental project	Results	Experimental project	Results
Catalase test	−	Fermented glucose-produced acid	+
Starch hydrolysis test	−	Fermented glucose-produced gas	−
Methyl red test	+	Lactose	+
Enzyme hydrolysis test	−	Maltose	+
pH 4.5 growth test	+	Mannitol	+
15°C growth test	+	Anaerobic nitrate gas production test	−
45°C growth test	+		

**FIGURE 2:**
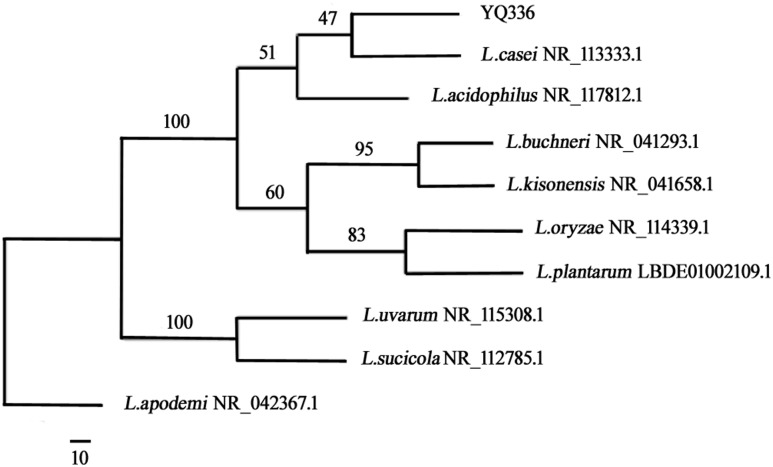
Phylogenetic relationships of YQ336 with related species based on partial 16S rDNA gene-sequence analysis. The phylogenetic tree was constructed using the neighbor-joining method in PHYLIP. The numbers at the nodes represent bootstrap confidence levels (expressed as the percentage) from 1,000 replicates. Reference sequences were obtained from the GenBank nucleotide sequence database.

A phylogenetic tree was generated using the neighbor-joining method after aligning the nucleotide sequences of strain YQ336 with sequences in the GenBank database (Figure 2). YQ336 formed a distinct cluster with *L. casei*, as supported by a bootstrap value of 47%. Therefore, YQ336 was identified as *L. casei* based on its colony behavior, morphological, and physiological characteristics, along with 16S rDNA sequence homology; accordingly, it was named *L. casei* YQ336.

### Growth and Acid Production Curve of *L. casei* YQ336

**FIGURE 3:**
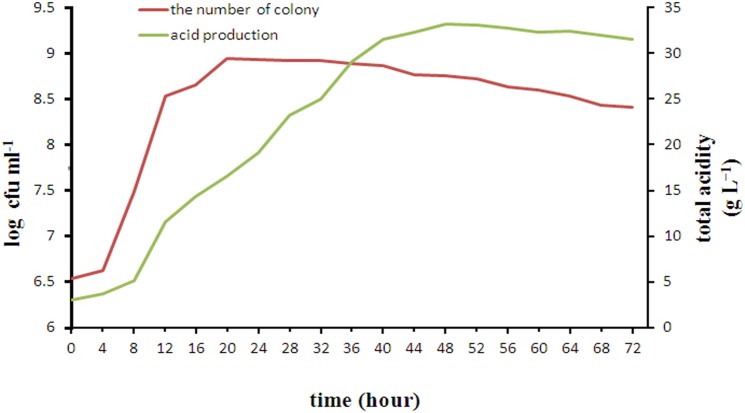
Growth and acid production curve of *L. casei* YQ336.

The changes in growth and acid production during 72 h incubation of *L. casei* YQ336 are shown in Figure 3. *L. casei* YQ336 entered the logarithmic growth phase after 4 h, the stable phase after 22 h, and the decay phase after 32 h. Although *L. casei* YQ336 entered the decay phase after 32 h, with the total number of live cells subsequently decreasing, the total acid amount of the fermentation broth continued to increase, reaching its highest value at 48 h of 33.165 g L^−1^, and the pH continued to decrease. This indicated that the bacterial growth curve was inconsistent with the metabolic acid production curve.

It was reported that *L. acidophilus*, *L. plantarum*, *L amylolyticus*, and *L. mesenteroides* had also been separated and isolated from natural fermented acid slurry (Qiao et al., 2010). However, to date these strains have not been used for industrial fermentation of tofu acid slurry. This study reports the first isolation of *L. casei* from naturally fermented acid slurry. Compared with other lactic acid bacteria isolated from naturally fermented acid slurry, YQ336 can not only produce a large amount of organic acid in the medium of soy whey with only a moderate amount of added carbon source (glucose), but also constitutes a type of probiotics allowed in food. This provides strong evidence for the safe production of acid slurry tofu in addition to supporting YQ336 as a potential strain for the development of probiotic beverages for human or animal use based on soy whey (Corzo-martínez et al., 2016; Li et al., 2017).

### Effect of Physical and Chemical Treatment of Acid Slurry on Its Coagulation Ability

Tofu is essentially a water-based protein gel that is formed by adding a coagulant to soy milk and heating, causing proteins to denature, dissociate, and then aggregate (Nguyen, 2014; Obiegbuna et al., 2014). Coagulation, which can be induced by acid, salt, or enzymes (Kohyama et al., 1995; Obatolu, 2008; Ono, 2008; Murekatete et al., 2014b; Zhu et al., 2016; Zheng et al., 2018), constitutes the most important step in the tofu making process. To study the coagulation mechanism of tofu by acid slurry, the effect of physical and chemical treatment on the coagulation of tofu was therefore studied.

**Table 3:** Coagulation results of different coagulants.

Physical or chemical treatments	pH of coagulant	Coagulation	pH of soy milk after adding acid slurry
Acid slurry (Blank control)	3.60 ± 0.026^a^	+	5.82 ± 0.066^a^
Heating	3.60 ± 0.040^a^	+	5.77 ± 0.045^a^
pH	7.00 ± 0.000^c^	−	6.73 ± 0.042^c^
Supernatant (centrifugal treatment)	3.60 ± 0.025^a^	+	5.84 ± 0.05^a^
Cell pellet (centrifugal treatment)	6.30 ± 0.010^b^	−	6.43 ± 0.057^b^

*Results are given as the means ± SD of triplicate determinations. Values with different small letters within the same column are significantly different (p < 0.05). “+” indicates soy protein coagulation. “−” indicates that soy protein did not coagulate.*

**Table 4:** HPLC analysis of *L. casei* YQ336 strain fermentation for different periods.

Fermentation time (h)	Tartaric acid (g/L)	Oxalic acid (g/L)	Lactic acid (g/L)	Citric acid (g/L)	Succinic acid (g/L)	Fumaric acid (g/L)	Total acid (g/L)	Lactic acid percentage of total acid (%)
0	0.22 ± 0.01^b^	0.25 ± 0.01^c^	1.79 ± 0.03^a^	1.09 ± 0.02^b^	0.16 ± 0.01^a^	0.0057 ± 0.00058^a^	3.51 ± 0.015^a^	50.85 ± 0.015^a^
8	0.20 ± 0.03^b^	0.25 ± 0.01^c^	3.39 ± 0.04^b^	0.92 ± 0.04^a^	0.18 ± 0.03^a^	0.0053 ± 0.00058^a^	4.95 ± 0.010^b^	68.48 ± 0.006^b^
16	0.16 ± 0.02^a^	0.24 ± 0.01^c^	11.57 ± 0.03^c^	1.19 ± 0.08^c^	0.43 ± 0.07^b^	0.0037 ± 0.00058^a^	13.59 ± 0.031^c^	85.13 ± 0.015^c^
24	0.17 ± 0.01^a^	0.25 ± 0.01^c^	16.71 ± 0.05^d^	1.20 ± 0.04^c^	0.68 ± 0.05^c^	0.003 ± 0.001^a^	19.00 ± 0.012^d^	87.90 ± 0.015^j^
32	0.19 ± 0.01^b^	0.27 ± 0.02^d^	21.85 ± 0.43^e^	1.73 ± 0.05^d^	0.95 ± 0.05^d^	0.009 ± 0.0036^b^	24.99 ± 0.025^e^	87.54 ± 0.010^f^
40	0.31 ± 0.01^c^	0.30 ± 0.01^e^	26.99 ± 0.06^f^	2.26 ± 0.06^h^	0.96 ± 0.03^d^	0.011 ± 0.003^c^	30.83 ± 0.025^f^	87.42 ± 0.015^h^
48	0.38 ± 0.02^d^	0.33 ± 0.02^f^	28.81 ± 0.03^i^	2.27 ± 0.03^h^	1.18 ± 0.05^e^	0.015 ± 0.003^d^	32.99 ± 0.031^j^	87.32 ± 0.006^e^
56	0.53 ± 0.03^e^	0.12 ± 0.01^b^	28.67 ± 0.04^i^	2.12 ± 0.04^g^	1.30 ± 0.08^f^	0.015 ± 0.0025^d^	32.76 ± 0.031^i^	87.53 ± 0.015^g^
64	0.59 ± 0.02^f^	0.11 ± 0.01^b^	27.82 ± 0.03^h^	2.03 ± 0.04^f^	1.42 ± 0.04^g^	0.011 ± 0.0021^c^	31.59 ± 0.332^h^	86.98 ± 0.015^d^
72	0.62 ± 0.02^g^	0.05 ± 0.01^a^	27.52 ± 0.11^g^	1.93 ± 0.05^e^	1.27 ± 0.06^f^	0.0097 ± 0.0031^b^	31.39 ± 0.015^g^	87.64 ± 0.010^i^

*Results are given as the means ± SD of triplicate determinations. Values with different small letters within the same column are significantly different (p < 0.05).*

The acid slurry fermented by *L. casei* YQ336 was, respectively, heated, adjusted to pH, and centrifuged to determine the effect of different treatments on the coagulation ability of the acid slurry for soy protein. The results are shown in Table 3. After the acid slurry was centrifuged at 10,000 × *g*, only the supernatant, but not the precipitate, was able to coagulate soy protein, indicating that the coagulant did not comprise the protein distributed on the surface of *L. casei* YQ336 cells, but rather a metabolite of *L. casei* YQ336 in the fermentation broth. Moreover, heat-enzyme treatment had no effect on the coagulation of acid slurry, which indicated that the enzymes among the metabolites of *L. casei* YQ336 did not represent the main factors for the coagulation. However, acid slurry did not induce soy protein coagulation when its pH of was adjusted to 7, which implied that the acid in acid slurry played an important in the tofu coagulation.

**Table 5:** Coagulation results of the simulation solutions.

Coagulant	pH of coagulant	Coagulation	pH after adding coagulant
SS1 (containing lactic acid and metal ions)	3.60 ± 0.036^b^	+	5.83 ± 0.08^a^
SS2 (containing only metal ions)	7.00 ± 0.000^c^	−	6.78 ± 0.046^b^
SS3 (containing only lactic acid)	2.29 ± 0.015^a^	+	5.76 ± 0.046^a^

*Results are given as the means ± SD of triplicate determinations. Values with different small letters within the same column are significantly different (p < 0.05). SS, simulated solution. + indicates soy protein coagulation. - indicates that soy protein did not coagulate.*

### Composition of Acid Slurry Fermented by *L. casei* YQ336

The composition of acid slurry fermented by *L. casei* YQ336 was determined. The moisture, total acid, protein, ash, fat, and soluble solids were 95.51 ± 1.10, 1.92 ± 0.06, 0.94 ± 0.19, 0.27 ± 0.05, 0.13 ± 0.02, and 3.48 ± 0.03%, respectively. Salts and organic acids were present in the acid slurry that might play a role in the coagulation of tofu. Therefore, the types and contents of metal ions and organic acids in acid slurry were further analyzed. Four types of metal ion; i.e., Ca^2+^, Mg^2+^, Na^+^, and K^+^ were effectively detected in acid slurry by inductively coupled plasma spectrometry, showing contents of 0.63 ± 0.036, 0.17 ± 0.017, 1.33 ± 0.04, and 2.06 ± 0.062 g L^−1^, respectively. In turn, the types and contents of organic acids in the *L. casei* YQ336 fermented acid slurry were determined by HPLC (Table 4). Six types of organic acids; i.e., tartaric, oxalic, lactic, citric, succinic, and fumaric acids were separated and effectively detected during the acid slurry fermentation by *L. casei* YQ336, although acetic acid was not detected. Lactic acid exhibited the highest content and increased to a maximum of 28.81 ± 0.032 g L^−1^ at 48 h of fermentation, then subsequently stabilized.

**Table 6:** Textural properties of tofu produced with different coagulants.

Coagulant	Hardness (N)	Elasticity (mm)	Adhesive (N)	Chewability (mJ)	Sensory score (100)
AS	14.43 ± 0.47^a^	4.24 ± 0.046^b^	8.43 ± 0.25^a^	39.85 ± 0.91^a^	92.3 ± 1.89^c^
SS1 (containing lactic acid and metal ions)	14.63 ± 0.61^a^	4.11 ± 0.085^b^	8.33 ± 0.55^a^	40.96 ± 0.76^a^	90.5 ± 1.58^b^
SS3 (containing only lactic acid)	16.33 ± 0.40^b^	3.83 ± 0.071^a^	11.37 ± 0.59^b^	48.36 ± 1.60^b^	88.5 ± 1.58^a^

*Results are given as the means ± SD of triplicate determinations. Values with different small letters within the same column are significantly different (p < 0.05). AS, acid slurry fermented by L. casei YQ336. SS, simulated solution.*

### Effect of Soy Protein Coagulation by the Simulated Acid Slurry

Simulated solutions were prepared according to the content of lactic acid and metal ions in the acid slurry fermented by *L. casei* YQ336, and the coagulation abilities of the three simulated solutions on soy protein were detected (Table 5). Both SS1 (containing both lactic acid and metal ions) and SS3 (only containing lactic acid) were able to coagulate soy protein, whereas SS2 (only containing metal ions) could not. This suggested that the lactic acid in acid slurry comprised the main factor for coagulating soy protein, rather than the metal ions.

### Texture Properties of Tofu Coagulated by Simulated Acid Slurry

To determine whether the metal ions in the acid slurry play a role in the coagulation process of soy protein, the texture properties of tofu coagulated by different coagulants were compared (Table 6). The texture of tofu coagulated by SS1 and AS was significantly better than that of SS3. The difference in the tofu texture between SS1 and the AS was not significant, although the AS tofu sensory score was better than that of SS1. Together, these results suggested that the metal ions contained in the acid slurry were not sufficient to coagulate soy protein, but were able to promote the lactic acid-mediated coagulation of soy protein and enhance the texture properties of the tofu.

### Microscopic Observation of Tofu

**FIGURE 4:**
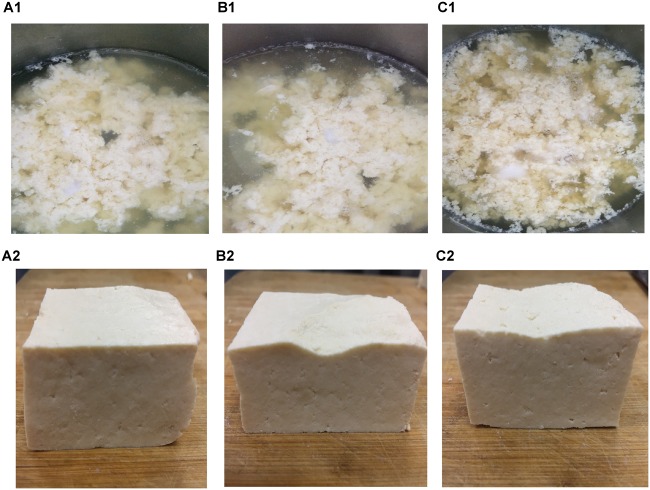
Soy milk and tofu prepared with different coagulants. **(A1, A2)** Acid slurry fermented by *L. casei* YQ336 (AS); **(B1,B2)** simulated solution 1, containing lactic acid and metal ions (SS1); and **(C1,C2)** simulated solution 3, containing only lactic acid (SS3).

**FIGURE 5:**
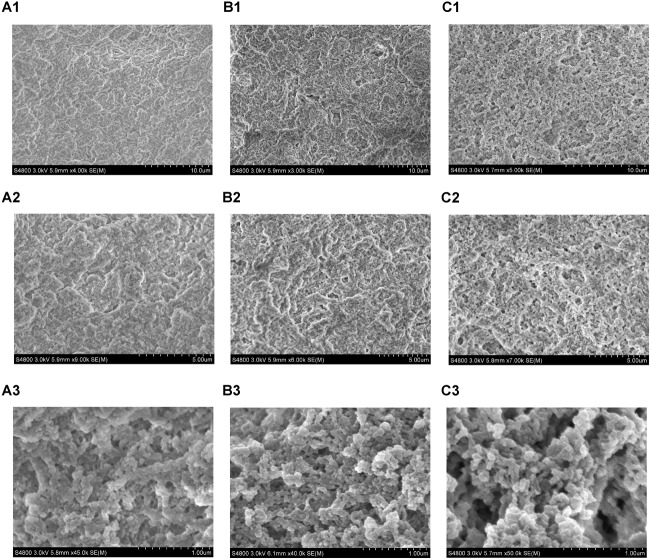
SEM micrographs of tofu prepared with different coagulants. **(A1–A3)** Acid slurry fermented by *L. casei* YQ336 (AS); **(B1–B3)** simulated solution 1, containing lactic acid and metal ions (SS1); and **(C1–C3)** simulated solution 3, containing only lactic acid (SS3).

The microstructure of the tofu coagulated by SS1, SS3, and AS was compared. The soy protein coagulation and tofu are shown in Figure 4; the SEM images are shown in Figure 5. The soy protein coagulated by AS yielded a large gel block, with the tofu exhibiting compact structure and small pores (Figure 4A1,A2); SEM displayed small microstructure and compact network structure (Figure 5A1–A3). The tofu coagulated by AS demonstrated the best tissue state among three coagulant-solidified tofu samples, followed by SS1-coagulated tofu (Figure 4B1,B2, 5B1–B3). The soy protein coagulated by SS3 (containing only lactic acid) exhibited small gel formation and a loose protein structure (Figure 4C1,C2), with SEM of the tofu displaying large pore structure and a loose network structure (Figure 5C1–C3).

Overall, the texture and microstructure of the tofu coagulated by SS1 were significantly better than those by SS3, indicating that the metal ions in the acid slurry could promote the coagulation of the tofu by the organic acids. In addition, these findings suggested that the enzyme produced by lactic acid bacteria also played a role in the coagulation of tofu, as the state and microstructure of the tofu coagulated by the acid slurry were better than those by SS1 and SS3.

Therefore, it was inferred that after the denaturation of soy protein by heating, the protein chain was unfolded, exposing hydrophobic region so that the protein molecule exhibited more negative charges (Kohyama et al., 1995). This generated a strong electrostatic repulsion between molecules, rendering the soy protein as difficult to approach and unable to coagulate (Murekatete et al., 2014b; Murekatete et al., 2015; Hsia et al., 2016). However, after the acid slurry was added, soy protein could coagulate under the influence of three factors. First, a large amount of organic acid in the acid slurry would hydrolyse to generate hydrogen ions, so that the negative charge of the soy protein was reduced and the electrostatic repulsion between molecules was weakened. When the pH of the gel was reduced to the isoelectric point of the soy protein, the soy protein coagulated to reach the maximum gel strength (Murekatete et al., 2014b; Rui et al., 2016). Secondly, metal ions in acid slurry would react with phytic acid (salt) and citric acid (salt) in soy protein to further promote the pH reduction. Moreover, the positive ions of the salt would mask part of the negative charge of the protein, to further promote soy protein coagulation (Murekatete et al., 2014a; Li et al., 2015). Thirdly, the protease produced by the lactic acid bacteria in the acid slurry might partially hydrolyse soy protein, resulting in increased hydrophobicity of the protein surface, to further facilitate soy protein coagulation (Qiao and Li, 2007; Wei et al., 2018; Zheng et al., 2018). In summary, the organic acid in the acid slurry appeared to be the main factor that promoted the coagulation of soy protein, with the metal ions and the enzymes produced by the lactic acid bacteria promoting the organic acid-mediated soy protein coagulation.

## Conclusion

Acid slurry has been used to coagulate tofu in China for over four centuries. Here, *L. casei* YQ336 was isolated as the dominant genus for coagulating tofu and its coagulation mechanism was investigated. The hydrogen ions produced by organic acids in the acid slurry cause electrostatic repulsion between soy protein molecules to weaken, comprising the main factor in tofu coagulation. The metal ions further decrease soy milk pH and the protease partially hydrolyses soy protein, serving as secondary factors promoting soy protein coagulation. This study provides a basic theory and technical references for the industrial production of acid slurry tofu.

## Author Contributions

LZ, QY, YX, YY, and XL performed all experiments and wrote the manuscript. YY, HZ, and ZZ conducted the experiments and data analysis. All authors read and approved the manuscript.

## Conflict of Interest Statement

The authors declare that the research was conducted in the absence of any commercial or financial relationships that could be construed as a potential conflict of interest.

## Footnotes

1 http://ccb.jhu.edu/software/FLASH/

2 http://www.mothur.org/

3 http://qiime.org/scripts/pick_otus.html

4 http://greengenes.secondgenome.com/

5 www.ncbi.nlm.nih.gov/blast

## Supplementary Material

FIGURE S1 Pie charts showing the abundance of *Lactobacillus* oligotypes in different samples. Oligotypes with abundance <2% in all samples are summed up as “others”. Average values for the three replicates are reported.

TABLE S1 Tofu sensory evaluation.
