# Supplementary figures and images for: Naturally Fermented Acid Slurry of Soy Whey: High-Throughput Sequencing-Based Characterization of Microbial Flora and Mechanism of Tofu Coagulation
Source: Front Microbiol. 2019 May 14;10:1088. doi: 10.3389/fmicb.2019.01088 (PMC6527785; doi:10.3389/fmicb.2019.01088)

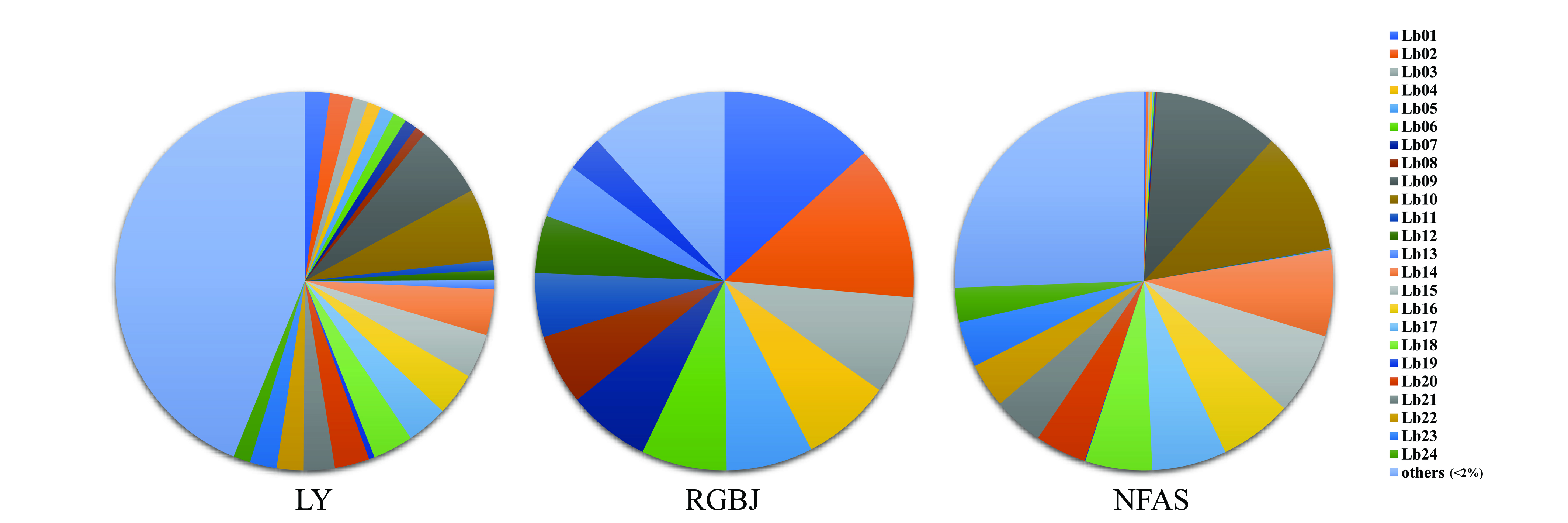

Supplement: FIGURE S1 — Pie charts showing the abundance of Lactobacillus oligotypes in different samples. Oligotypes with abundance <2% in all samples are summed up as “others”. Average values for the three replicates are reported. [file Image_1.TIF]
