# Supplementary material for: Naturally Fermented Acid Slurry of Soy Whey: High-Throughput Sequencing-Based Characterization of Microbial Flora and Mechanism of Tofu Coagulation
Source: Front Microbiol. 2019 May 14;10:1088. doi: 10.3389/fmicb.2019.01088 (PMC6527785; doi:10.3389/fmicb.2019.01088)
Supplement: TABLE S1 — Tofu sensory evaluation. [file Table_1.DOC]

**Supplementary data**

| **TABLE S1 | Tofu sensory evaluation** | | |
| --- | --- | --- |
| Evaluation index | Rating basis | Score |
| Colour  (20 points) | White  Milky white  Light yellow | 16–20  11–15  1–10 |
| Flavour  (20 points) | Strong tofu flavour and no other odour  Tofu flavour but slightly sour  Tofu flavour but a big sour taste  Odour other than tofu | 16–20  11–15  6–10  1–5 |
| Organizational status  (30 points) | Block shape, suitable soft and hard, fine texture, flexible, non-stick surface  Block shape, suitable soft and hard, slightly fine texture, slightly flexible, non-stick surface  Block shape, uncomfortable hardness, not good texture, small elasticity, non-stick surface  Incomplete block shape, uncomfortable hardness, not good texture, small elasticity, sticky surface | 26–30  21–25  11–20  1–10 |
| Taste  (15 points) | Delicate and smooth, good elasticity, no sour taste  Smooth and slippery, slightly elasticity, slightly sour  Coarse or paste, inelastic, sour, odour other than tofu | 11–15  6–10  1–5 |
| Section structure  (15 points) | The section is very neat and the surface is very smooth  The section is neat and the surface is smooth  Irregular section and rough surface | 11–15  6–10  1–5 |
